# Supplementary material for: Random terpolymer based on thiophene-thiazolothiazole unit enabling efficient non-fullerene organic solar cells
Source: Nat Commun. 2020 Sep 14;11:4612. doi: 10.1038/s41467-020-18378-9 (PMC7490407; doi:10.1038/s41467-020-18378-9)
Supplement: Supplementary file 1 — Supplementary Information [file 41467_2020_18378_MOESM1_ESM.pdf]

SUPPLEMENTARY INFORMATION

Random Terpolymer Based on Thiophene-Thiazolothiazole Unit Enabling Efficient Non-Fullerene Organic Solar Cells

Jingnan Wu[1], Guangwei Li[1], Jin Fang[1], Xia Guo[1], Lei Zhu[2], Bing Guo[1], Yulong Wang[1], Guangye Zhang[3], Lingeswaran Arunagiri[4], Feng Liu[2], He Yan[*4], Maojie Zhang[*1], Yongfang Li[1,5]

[1] Laboratory of Advanced Optoelectronic Materials, College of Chemistry, Chemical Engineering and Materials Science, Soochow University, Suzhou 215123, China.
[2] Department of Physics and Astronomy and Collaborative Innovation Center of IFSA (CICIFSA), Shanghai Jiao Tong University, Shanghai 200240, China.
[3] Flat/RM B, 12/F, Hang Seng Causeway Bay BLDG, 28 Yee Wo Street, Causeway Bay, Hong Kong.
[4] Department of Chemistry and Hong Kong Branch of Chinese National Engineering Research Center for Tissue Restoration & Reconstruction, Hong Kong University of Science and Technology (HKUST), Clear Water Bay, Kowloon, Hong Kong, China.
[5] Beijing National Laboratory for Molecular Sciences, CAS Key Laboratory of Organic Solids, Institute of Chemistry, Chinese Academy of Sciences, Beijing 100190, China.

[*]Email: mjzhang@suda.edu.cn; hyan@ust.hk

Figure S.1 | Synthetic scheme for the polymers and chemical structure of Y6.

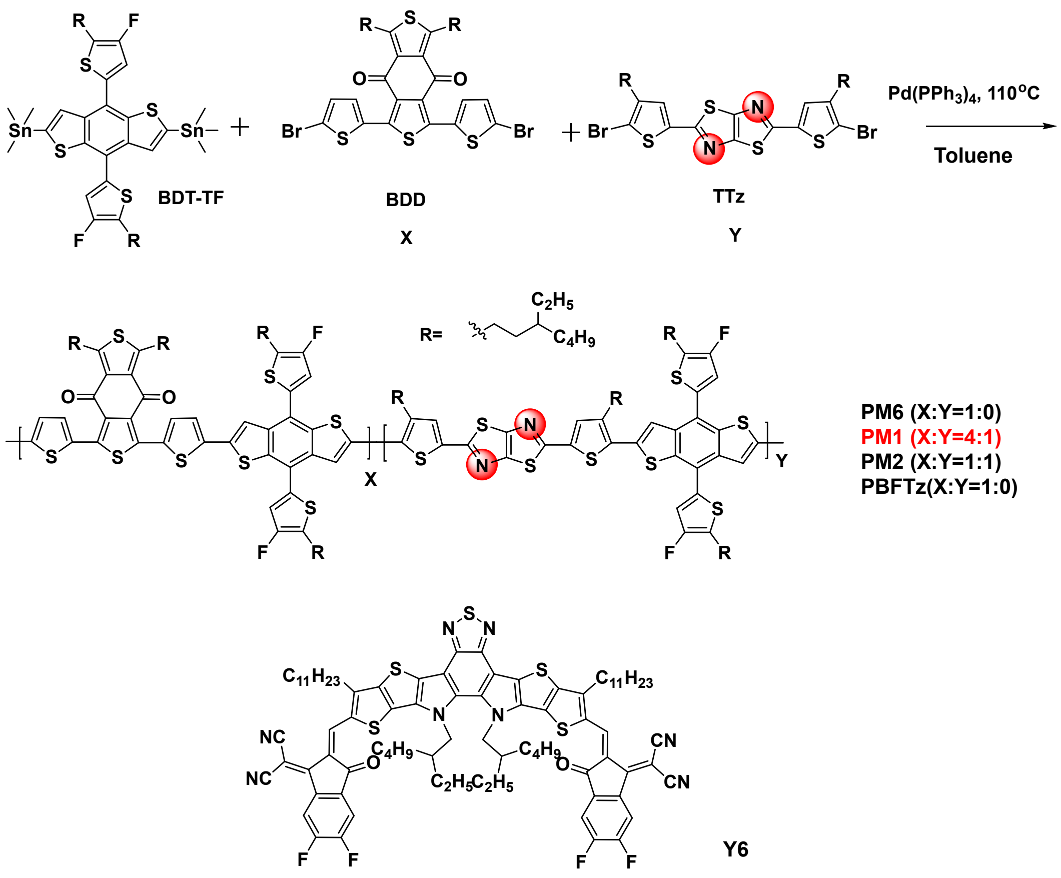

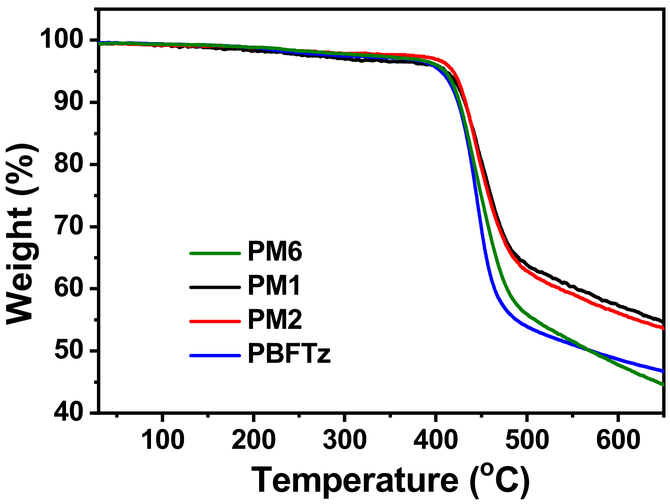

Figure S.2 | TGA analysis (heating ramp: 10 ºC min^{-1}) of polymers in nitrogen.

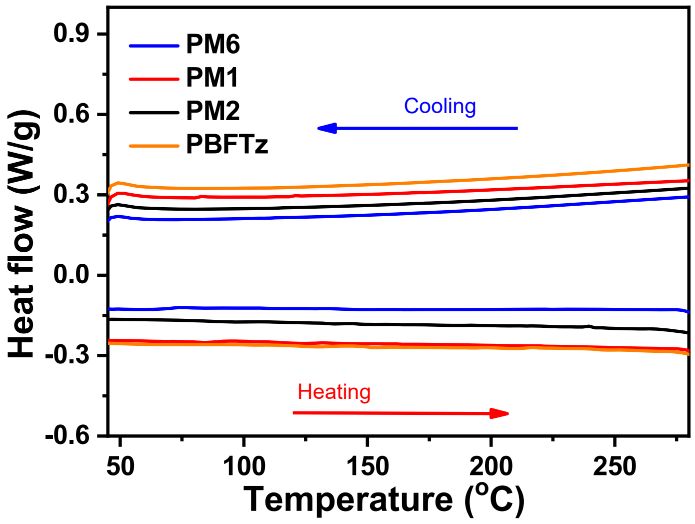

Figure S.3 | DSC curves of polymers at a scan rate of 10 ºC min^{-1} under nitrogen.

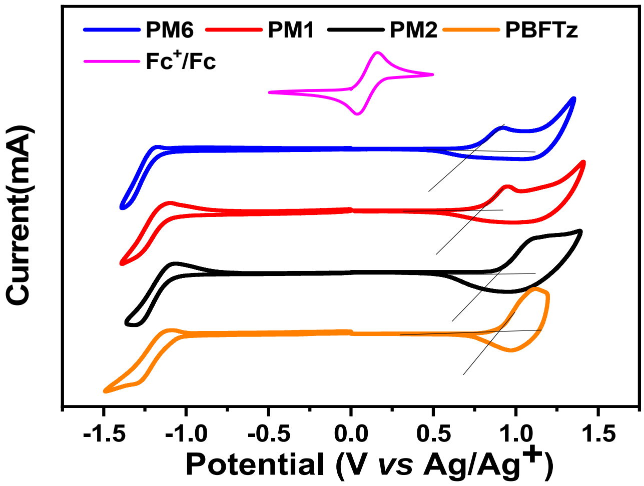

Figure S.4 | Cyclic voltammograms of polymers. Cyclic voltammetry (CV) was performed on a Zahner Zennium IM6 electrochemical workstation with a three-electrode system in 0.1 mol L-1 Bu_4NPF_6 acetonitrile solutions at a scan rate of 50 mV s^{-1}. The three-electrode system included glassy carbon disk, platinum wire and Ag/Ag+ electrode as the working electrode, counter electrode and reference electrode, respectively. The potential of the Ag/Ag+ reference electrode was internally calibrated by using the ferrocene/ferrocenium redox couple (Fc/Fc^+), and the Ag/Ag^+ reference electrode possessed an energy level of 4.73 eV.

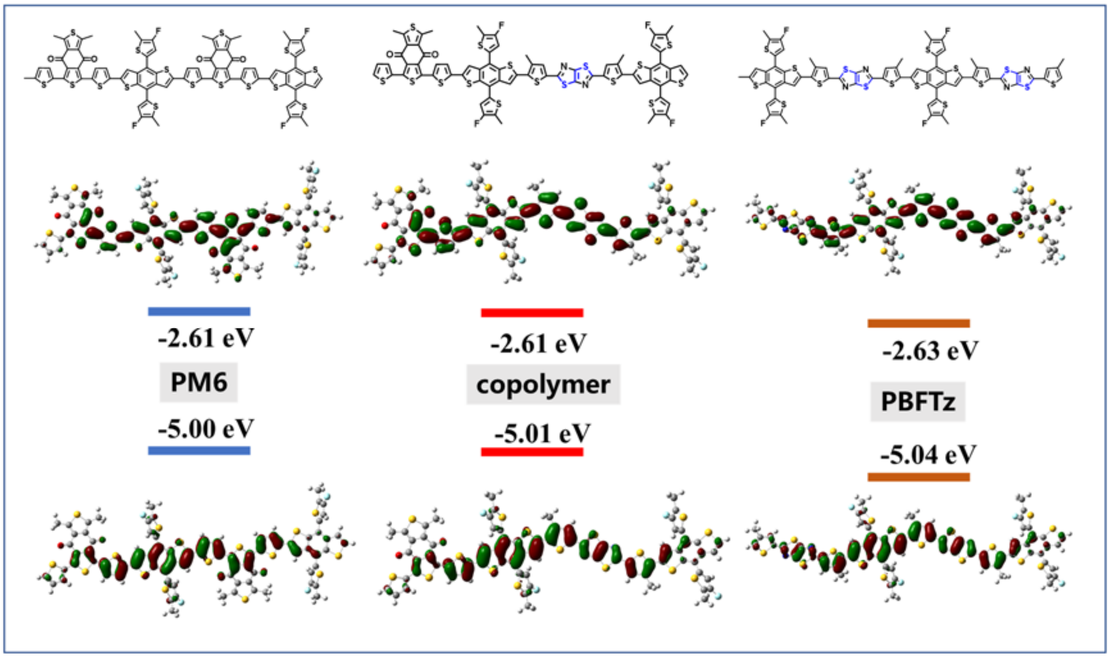

Figure S.5 | Molecular energy levels and wavefunction distributions of the frontier orbits for by polymer models calculated by DFT/B3LYP/6-31G (d, p) with methyl groups in replacing alkyl substituents to simplify the calculations.

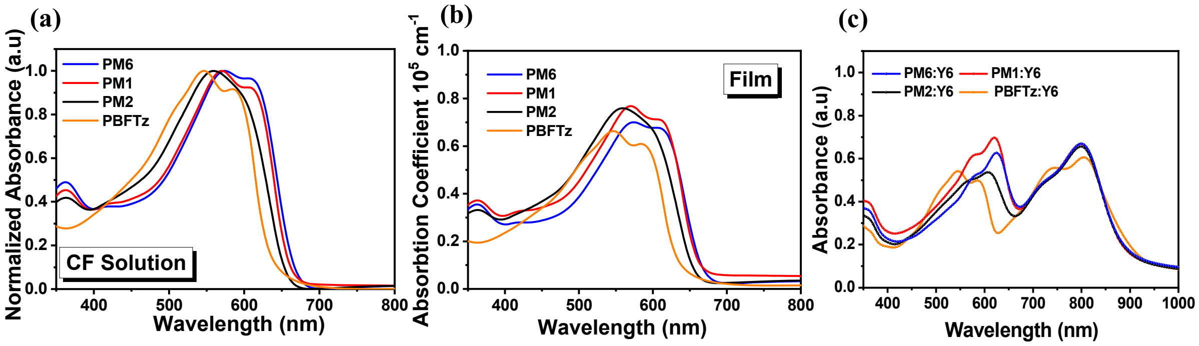

Figure S.6 | (a) Normalized absorption spectra of polymers in chloroform. (b) the absorption coefficients of neat polymer films (c) UV-vis absorption spectra of the polymer:Y6 blend films. UV-vis absorption spectrum was recorded on a UV-Vis-NIR Spectrophotometer of Agilent Technologies Cary Series, in which the extinction coefficient was defined by the absorption intensity of the active layer with a thick of 1 cm (ca. 100 nm for blends).

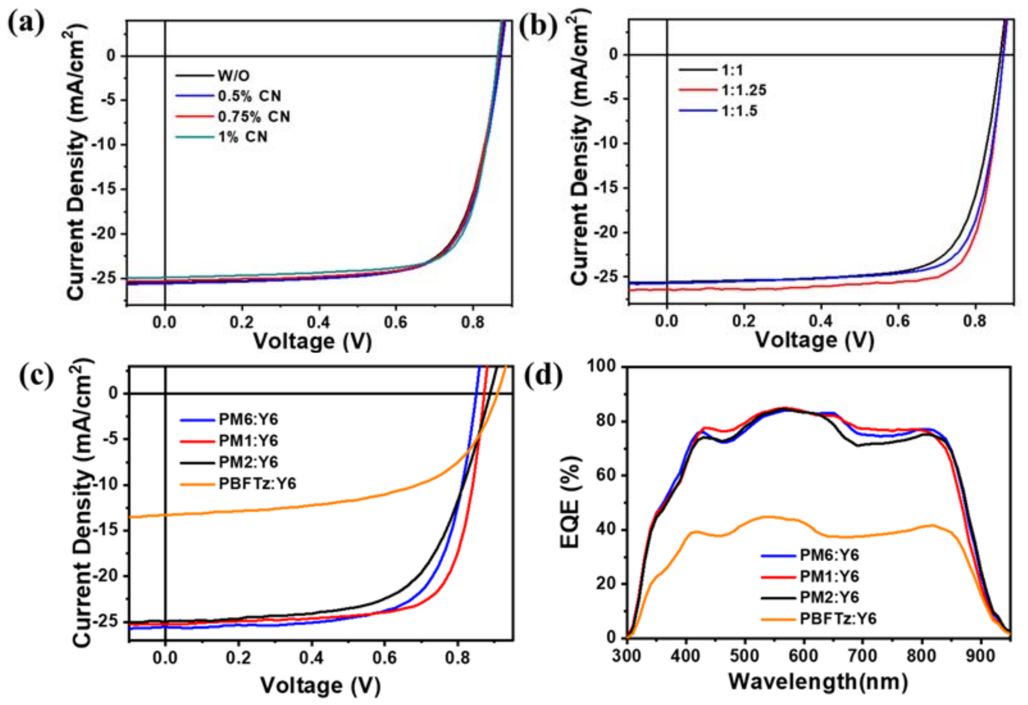

Figure S.7 | The *J-V* characteristics of (a) the OSCs based on PM1:Y6 at different additive. (b) the OSCs based on PM1:Y6 at different D/A weight ratios with additive (0.75 vol %); and (c) the OSCs based on as-cast device under the irridiation of AM 1.5G, 100 mW/cm^2. (d) the EQE spectra of the OSCs.

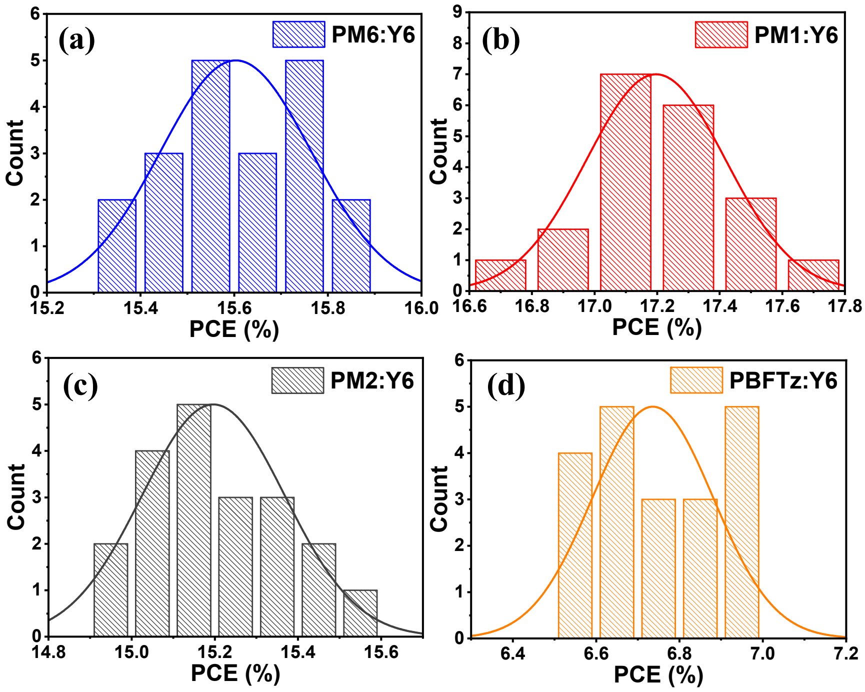

Figure S.8 | Statistical diagram of PCEs for 20 polymer:Y6-based cells.

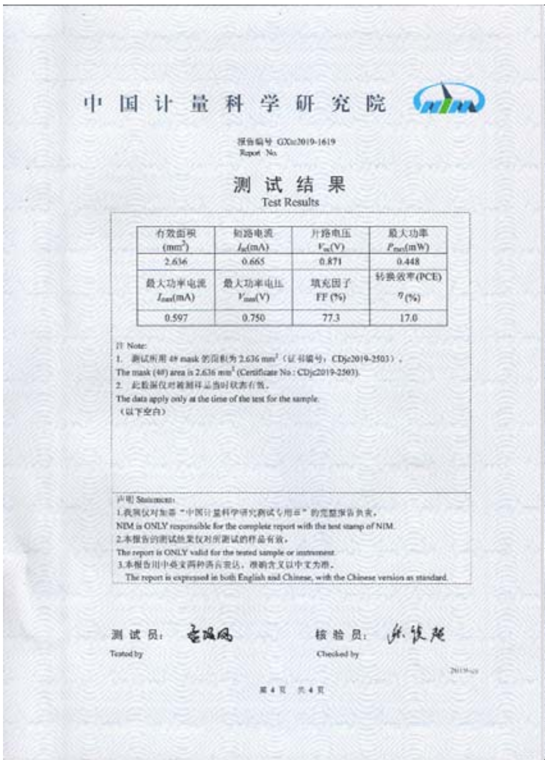

中 国 计 量 科 学 研 究 院

报告编号 GXtc2019-1619
Report No.

测 试 结 果
Test Results

有效面积 (mm²)	短路电流 I_{sc}(mA)	开路电压 V_{oc}(V)	最大功率 P_{max}(mW)
2.636	0.665	0.871	0.448
最大功率电流 I_{max}(mA)	最大功率电压 V_{max}(V)	填充因子 FF (%)	转换效率(PCE) η (%)
0.597	0.750	77.3	17.0

注 Note:
1. 测试所用 4# mask 的面积为 2.636 mm²（证书编号：CDjc2019-2503）。
The mask (4#) area is 2.636 mm² (Certificate No.: CDjc2019-2503).
2. 此数据仅对被测样品当时状态有效。
The data apply only at the time of the test for the sample.
（以下空白）

声明 Statement:
1.我院仅对加盖"中国计量科学研究院测试专用章"的完整报告负责。
NIM is ONLY responsible for the complete report with the test stamp of NIM.
2.本报告的测试结果仅对所测试的样品有效。
The report is ONLY valid for the tested sample or instrument.
3.本报告用中英文两种语言表达，准确含义以中文为准。
The report is expressed in both English and Chinese, with the Chinese version as standard.

测 试 员：
Tested by

核 验 员：
Checked by

第 4 页 共 4 页

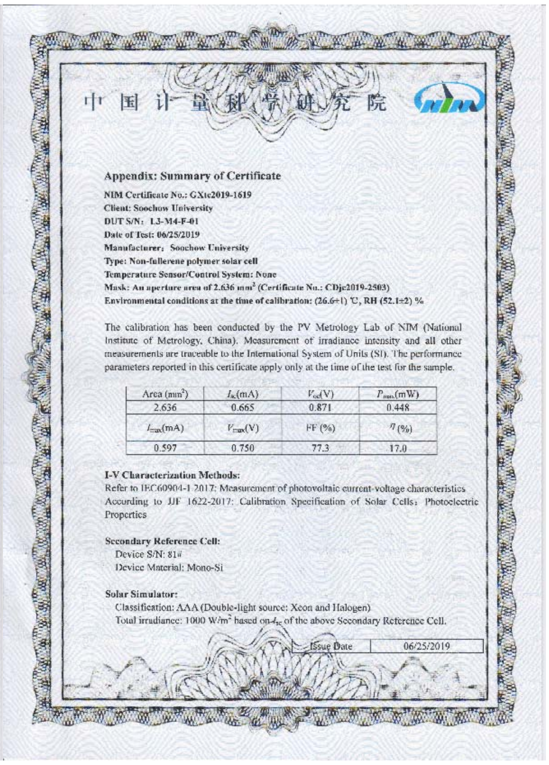

中 国 计 量 科 学 研 究 院

Appendix: Summary of Certificate

NIM Certificate No.: GXtc2019-1619
Client: Soochow University
DUT S/N: L3-M4-F-01
Date of Test: 06/25/2019
Manufacturer: Soochow University
Type: Non-fullerene polymer solar cell
Temperature Sensor/Control System: None
Mask: An aperture area of 2.636 mm² (Certificate No.: CDjc2019-2503)
Environmental conditions at the time of calibration: (26.6±1) ℃, RH (52.1±2) %

The calibration has been conducted by the PV Metrology Lab of NIM (National Institute of Metrology, China). Measurement of irradiance intensity and all other measurements are traceable to the International System of Units (SI). The performance parameters reported in this certificate apply only at the time of the test for the sample.

Area (mm²)	I_{sc}(mA)	V_{oc}(V)	P_{max}(mW)
2.636	0.665	0.871	0.448
I_{max}(mA)	V_{max}(V)	FF (%)	η (%)
0.597	0.750	77.3	17.0

I-V Characterization Methods:
Refer to IEC60904-1 2017: Measurement of photovoltaic current-voltage characteristics
According to JJF 1622-2017: Calibration Specification of Solar Cells: Photoelectric Properties

Secondary Reference Cell:
Device S/N: 81#
Device Material: Mono-Si

Solar Simulator:
Classification: AAA (Double-light source: Xeon and Halogen)
Total irradiance: 1000 W/m² based on I_{sc} of the above Secondary Reference Cell.

Issue Date	06/25/2019

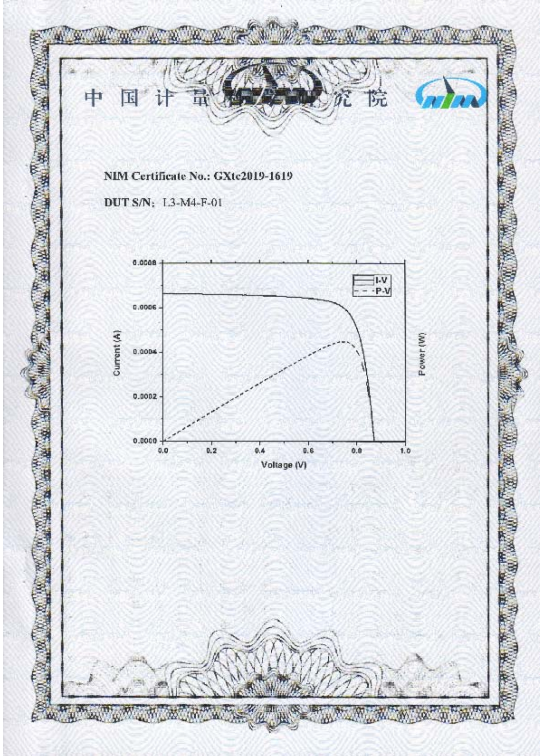

Figure S.9 | Test report of the PM1:Y6-based OSCs from National Institute of Metrology, China (NIM).

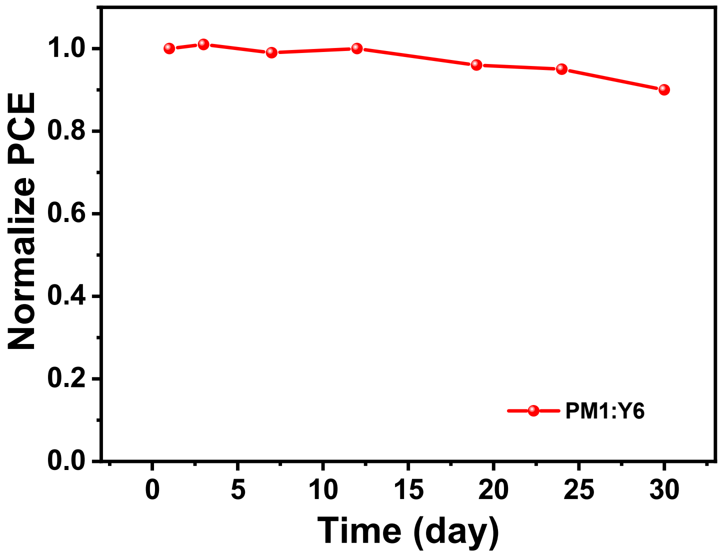

Figure S.10 | Storage stability of the optimized PM1:Y6 devices.

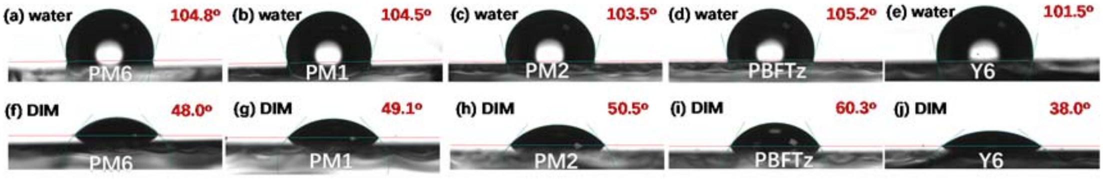

Figure S.11 | Water and diiodomethane (DIM) contact angles of the pristine polymers.

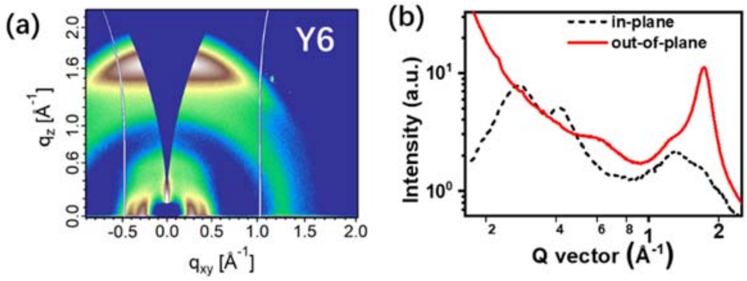

Figure S.12 | (a) 2D GIWAXS patterns for Y6 pure films. (b) Scattering profiles for pure films of Y6.

Grazing incidence wide-angle X-ray scattering measurements were performed at beamline 7.3.3 at the Advanced Light Source.1 Samples were prepared on Si substrates using identical blend solutions as those used in devices. The 10 keV X-ray beam was incident at a grazing angle of 0.12°-0.16°, selected to maximize the scattering intensity from the samples. The crystal coherence length (CCL) was defined as CCL = 0.9 × (2π/FWHM) (Å), where FWHM is the full width at half maximum of the corresponding diffraction peak.

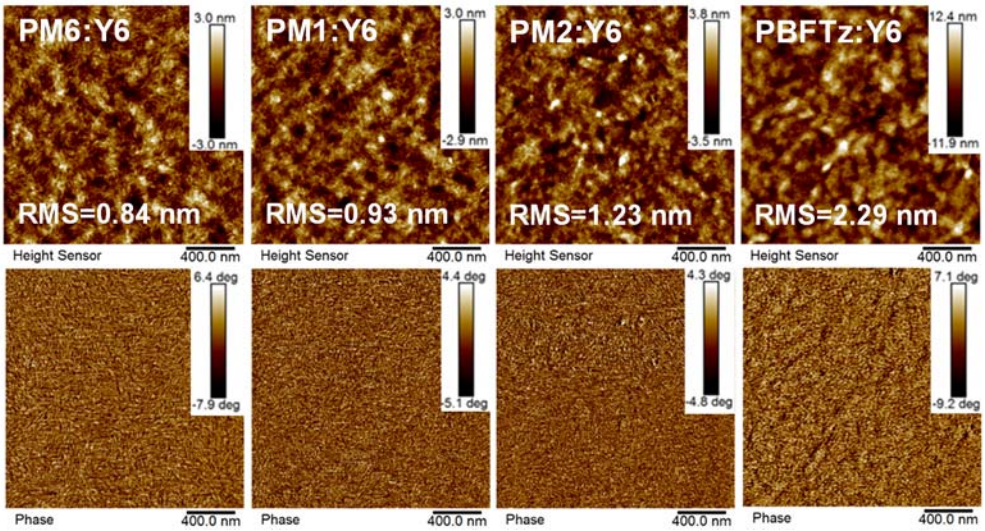

Figure S.13 | The AFM images of different blend films under the optimal conditions.

Atomic force microscopy (AFM) measurements were performed on a Dimension 3100 (Veeco) Atomic Force Microscope in the tapping mode.

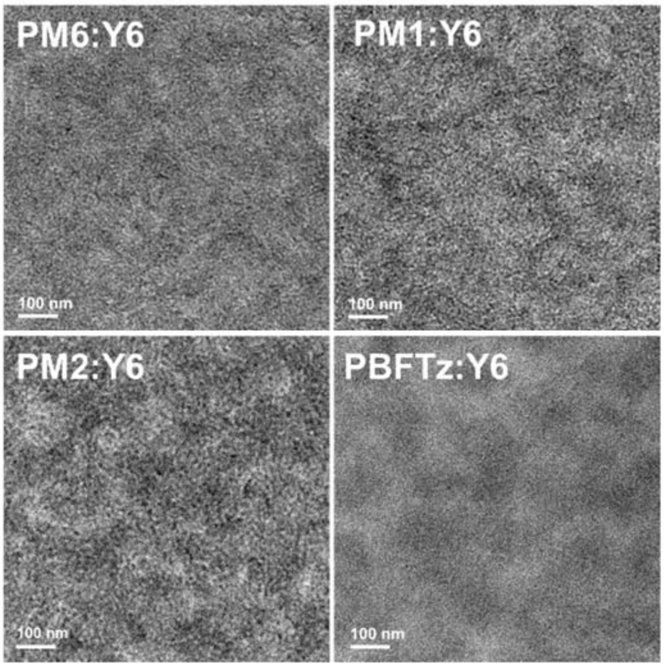

Figure S.14 | The TEM images of the polymer:Y6 blend films under the optimal conditions. Transmission electron microscopy (TEM) was performed using a Tecnai G2 F20 S-TWIN instrument at 200 kV accelerating voltage, where the polymer:Y6 films were prepared by the following processing techniques for the TEM measurement: The polymer:Y6 films were spin-cast on the ITO/PEDOT:PSS substrates, and then the resulting substrates with the polymer:Y6 films were submerged in deionized water to make these polymer:Y6 films float onto the water/air interface, and finally the floated polymer: Y6 films were picked up on unsupported 200 mesh copper grids.

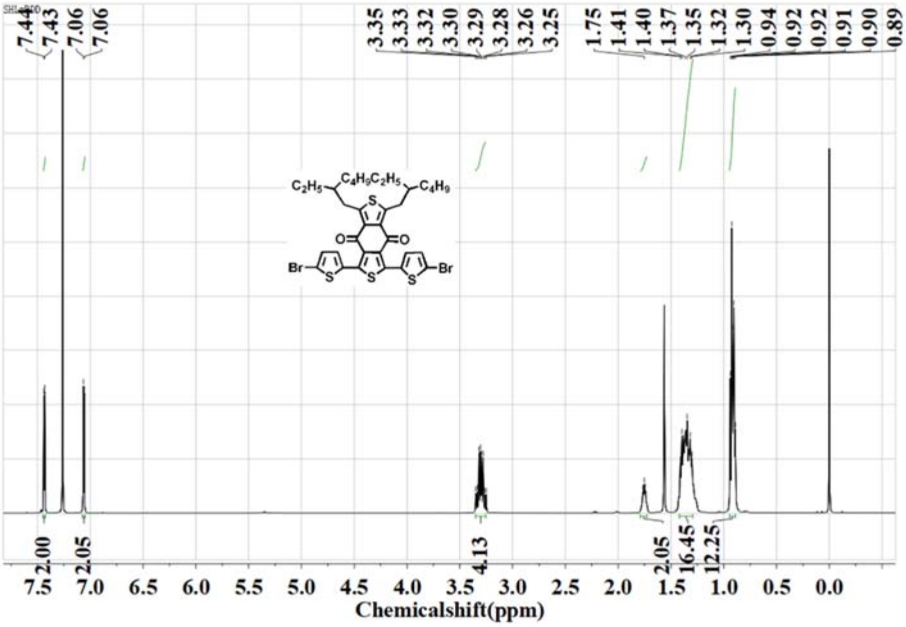

Figure S.15 | 1H NMR spectra of BDD unit.

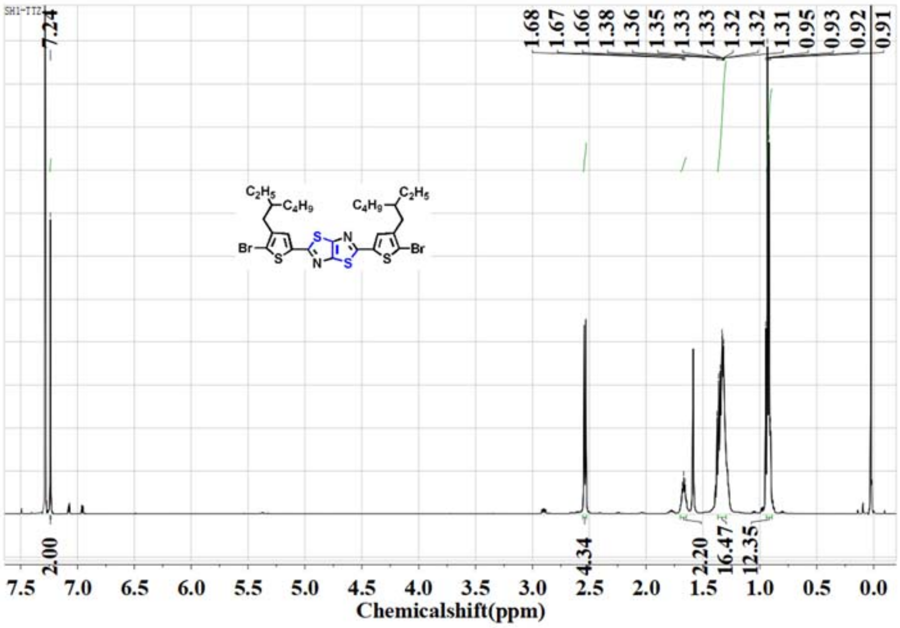

Figure S.16 | ^{1}H NMR spectra of TTz unit.

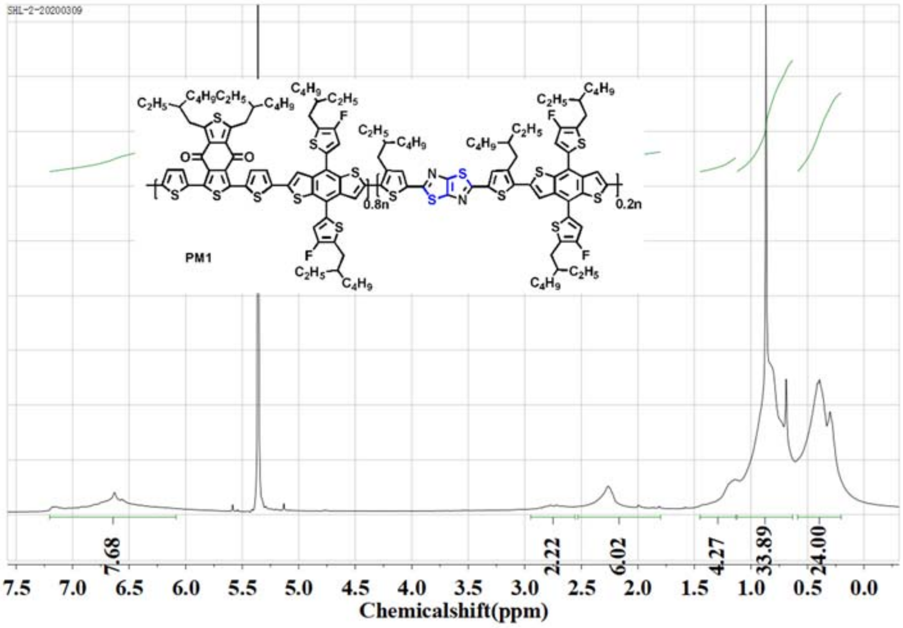

Figure S.17 | High-temperature (80ºC) ^{1}H NMR spectra of PM1.

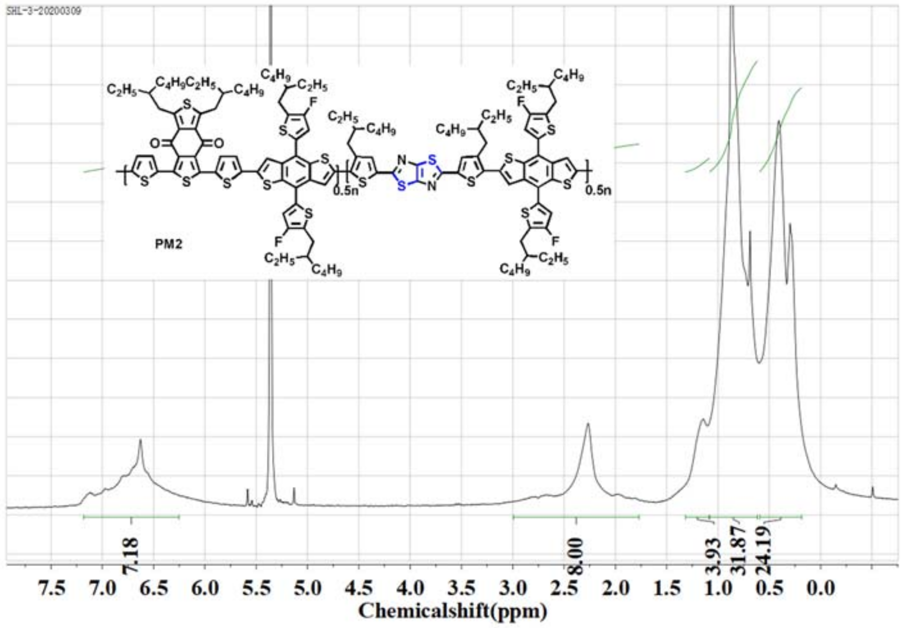

Figure S.18 | High-temperature (80ºC) ^{1}H NMR spectra of PM2.

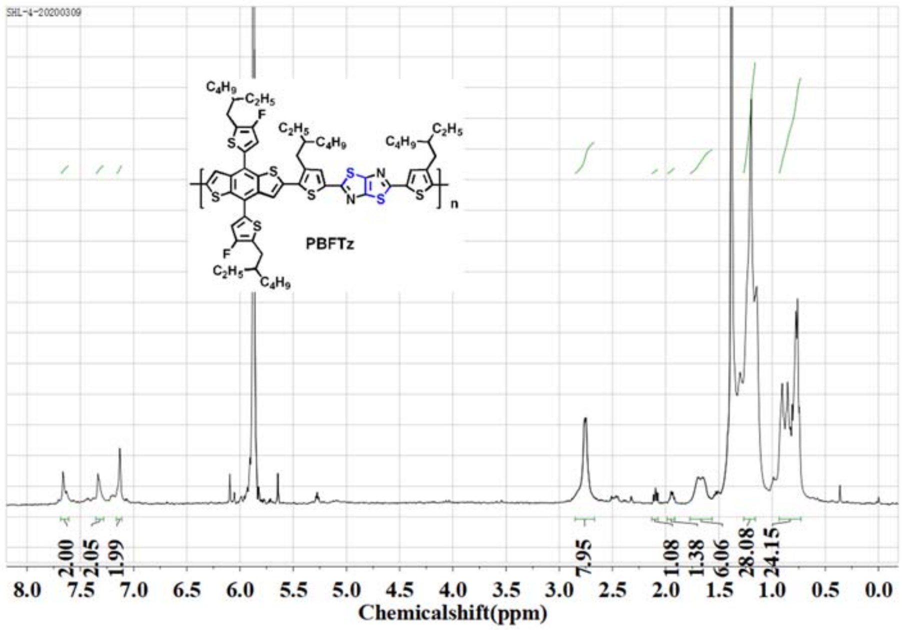

Figure S.19 | High-temperature (80°C) ^{1}H NMR spectra of PBFTz.

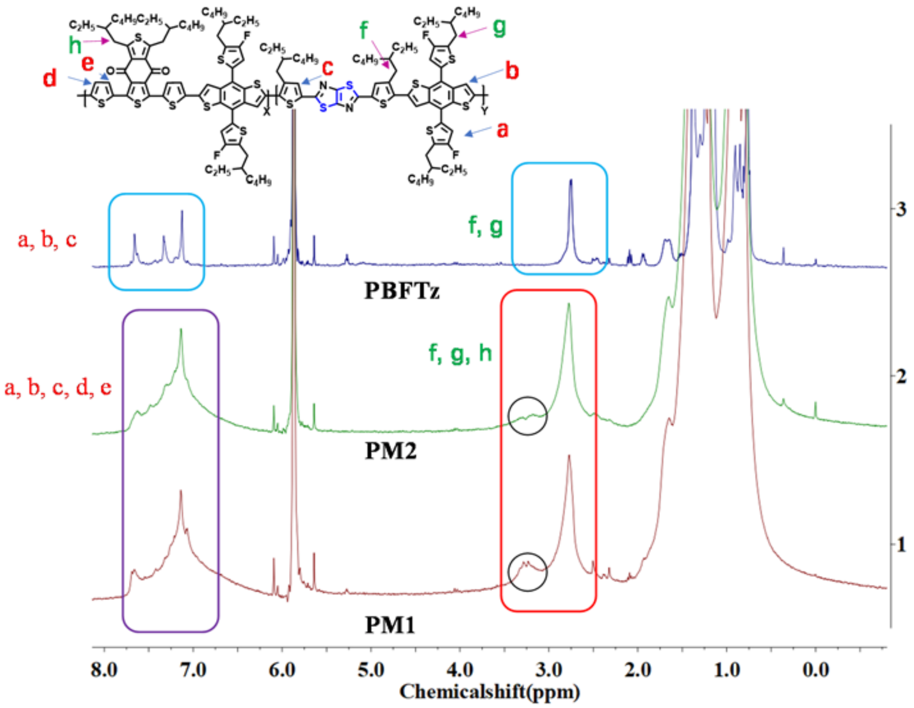

Figure S.20 | Comparison of ^{1}H NMR spectra of PM1(X:Y=4:1), PM2 (X:Y=1:1) and PBFTz.

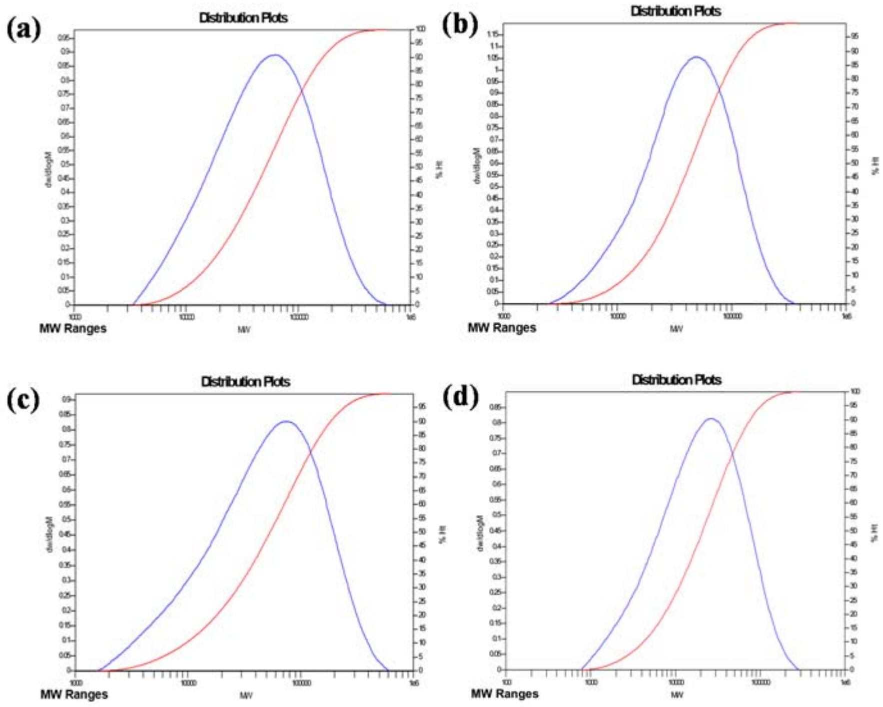

Figure S.21 | GPC traces of polymers: (a) PM6; (b) PM1; (c) PM2; (d) PBFTz. High temperature GPC with 1,2,4-trichlorobenzene as the eluent and polystyrene as a standard at 160 ^oC.

Table S1 | Basic properties of the polymers. The molecular weight of the polymer was measured by the GPC method with polystyrene as the standard and 1, 2, 4-tricholorobenzene as the solvent at 160 °C using Agilent Technologies PL-GPC220. Thermogravimetric analysis (TGA) was measured on TGA/DSC 3+ from METTLER TOLEDO using STARe Software at a heating rate of 10 °C min^{-1} under a nitrogen atmosphere.

**Polymer**	M_n **[kDa]**	**PDI**	E_g^{opt} **[eV]**[a)]	**HOMO [eV]**	**LUMO [eV]**	μ_h [b)] **(cm²·V⁻¹·S⁻¹) ×10⁴**
**PM6**	29.7	2.48	1.85	-5.48	-3.59	6.70 [6.13±0.42]
**PM1**	28.7	1.96	1.86	-5.50	-3.59	7.18 [7.02±0.16]
**PM2**	23.2	3.33	1.89	-5.60	-3.63	4.80 [4.38±0.35]
**PBFTz**	10.1	3.19	1.94	-5.63	-3.67	2.78 [2.41±0.29]

a) Calculated from the empirical formula: E^g_{opt} = 1240/ λ onset. b) the hole-only devices with the structure of ITO/PEDOT:PSS/polymer donor/MoO_3/Ag and the statistical values in square bracket are the average μ_h obtained from 4 $J^{1/2}$-V plots.

Table S2 | Photovoltaic performance parameters of the OSCs based on **PM1**:**Y6** at different additive, under the irridiation of AM 1.5G, 100 mW/cm^2.

**D/A (w/w)**	**CN**	V_{oc} **(V)**	J_{sc} **(mA/cm²)**	**FF (%)**	**PCE (%) Max./Avg.**
**1:1**	W/O	0.87	25.5	71.1	15.8/15.8
	0.5% CN	0.87	25.8	72.0	16.2/16.0
	0.75% CN	0.87	25.6	72.8	16.4/16.3
	1% CN	0.86	24.9	74.6	16.0/15.7

The integral J_{sc} from the EQE curves. The Average parameters were calculated from more than 20 independent cells.

Table S3 | Photovoltaic performance parameters of the OSCs based on **PM1**:**Y6** at different D/A weight ratios with additive (0.75 vol %), under the irridiation of AM 1.5G, 100 mW/cm^2.

D/A（w/w）	V_{oc} (V)	J_{sc} (mA/cm^2)	FF (%)	PCE (%) Max./Avg.
**1:1**	0.87	25.6	72.8	16.4/16.1
**1:1.25**	0.87	25.9	78.1	17.6/17.3
**1:1.5**	0.87	25.7	75.0	16.8/16.7

The integral J_{sc} from the EQE curves. The Average parameters were calculated from more than 20 independent cells.

Table S4 | Photovoltaic performance parameters of the OSCs with different polymers at D/A weight ratios (1:1.25, w/w) under the irridiation of AM 1.5G, 100 mW/cm^2.

Devices[a)]	V_{oc} (V)	J_{sc} (mA/cm^2)	FF (%)	Cal. J_{sc}[b)] (mA/cm^2)	PCE (%) Max./Avg.[c)]
**PM6 :Y6**	0.86	25.5	70	25.2	15.2 /15.0
**PM1 :Y6**	0.87	25.3	74	25.2	16.1 /15.8
**PM2 :Y6**	0.89	25.1	63	24.7	13.9 /13.6
**PBFTz:Y6**	0.91	13.3	57	12.6	6.9 /6.7

a) As-cast device. b) The integral J_{sc} from the EQE curves. c) The Average parameters were calculated from more than 20 independent cells.

Table S5. | Summary of the molecular weight of PM1/PM6 and photovoltaic performance of PM1/PM6-based single junction cells with different batches.

**Polymers**	**PM1**			**PM6**		
**Batches**	M_n [kDa]	**PDI**	**PCE$_{max}$ a)** [%]	M_n [kDa]	**PDI**	**PCE$_{max}$ a)** [%]
**Batch1**	21.2	2.2	17.1 [16.8]	12.2	3.5	15.2 [14.9]
**Batch2**	23.5	2.2	17.1 [16.9]	14.8	3.6	15.3 [15.1]
**Batch3**	24.1	2.2	17.3 [17.0]	21.4	3.0	16.5 [16.3]
**Batch4**	25.6	2.3	17.2 [17.0]	21.5	2.5	16.3 [16.2]
**Batch5**	26.7	2.1	17.5 [17.3]	26.2	2.2	15.7 [15.6]
**Batch6**	28.7	2.0	17.6 [17.3]	29.7	2.5	15.8 [15.6]
**Batch7**	31.5	2.3	17.0 [16.8]	38.9	2.1	15.5 [15.2]
**Batch8**	33.0	2.3	17.4 [17.0]	63.1	2.1	14.7 [14.4]

a) The PSCs based on polymer:Y6 (1:1.25) with 0.75% CN; the statistical values in square bracket are the average PCE obtained from 10 devices.

Table S6 | Optimal photovoltaic performance parameters of the OSCs with a device architecture of Glass/ITO/PEDOT:PSS/Active Layer/PFN-Br/Ag under the irridiation of AM 1.5G, 100 mW/cm^2.

**Active layer**	V_{oc} **(V)**	J_{sc} **(mA/cm^2)**	**FF (%)**	**PCE b) (%)**
**PM1:Y6**	0.87	25.9	78	17.6 [17.2±0.2]
**PM1:N3 a)**	0.88	25.8	75	17.0 [16.8±0.2]
**PM6:N3 c)**	0.84	25.8	74	16.0 [15.8±0.2]

a) 0.75% CN, 100°C+10min. b) The statistical values in square bracket are the average PCE obtained from 15 devices. c) Ref [1]

Table S7 | The surface energy and Flory–Huggins interaction parameter (χ) of the donor and acceptor materials.

**Donor**	γ_{donor} **(mJ/m^2)**	**Acceptor**	$\gamma_{acceptor}$ **(mJ/m^2)**	χ/ **K(γ_D^{-2}-γ_A^{-2})^2**	**FF**
**PM6**	38.12	Y6	43.82	0.199K	72
**PM1**	37.31	Y6	43.82	0.262K	78
**PM2**	36.12	Y6	43.82	0.372K	69
**PBFTz**	29.64	Y6	43.82	1.382K	59

Table S8 | Summary of the crystal coherence length and *d*-spacing of neat polymer films.

		**LOCATION (Å^{-1})**	**FWHM (Å^{-1})**	**CL (Å)**	**d-spacing (Å)**
**PM6**	OOP (010)	1.66	0.289	19.60	3.78
**PM1**		1.66	0.262	21.61	3.78
**PM2**		1.67	0.315	17.95	3.76
**PBFTz**		1.68	0.329	17.20	3.74
**PM6**	IP (100)	0.29	0.095	59.54	21.88
**PM1**		0.29	0.112	50.58	21.68
**PM2**		0.30	0.117	48.53	20.76
**PBFTz**		0.31	0.083	68.47	20.13

Table S9 | Summary of the crystal coherence length and *d*-spacing of blend films

		LOCATION (Å^{-1})	FWHM (Å^{-1})	CL (Å)	d-spacing (Å)	Hight
**PM6:Y6**	OOP (010)	1.72	0.214	26.48	3.65	307.24
**PM1:Y6**		1.72	0.218	26.00	3.65	408.48
**PM2:Y6**		1.72	0.235	24.04	3.65	366.87
**PBFTz:Y6**		1.73	0.216	26.11	3.64	318.24
**Y6**		1.73	0.210	26.87	3.63	
**PM6:Y6**	IP (100)	0.29	0.078	72.70	21.67	663.52
**PM1:Y6**		0.30	0.073	77.03	21.65	796.6
**PM2:Y6**		0.30	0.078	72.07	21.28	581.93
**PBFTz:Y6**		0.31	0.059	95.95	21.07	590.28
**Y6**		0.27	0.093	60.61	20.04	

Table S10 | Electron and hole SCLC mobilities of blend films with 0.75% CN treatment.

**Blend**[a)]	μ_h **(cm^2·V^{-1}·S^{-1})** ×10^4	μ_e **(cm^2·V^{-1}·S^{-1})** ×10^4	μ_e/μ_h
PM6:Y6	3.68 [3.42±0.29]	5.16 [4.63±0.51]	1.40
PM1:Y6	5.02 [4.69±0.53]	5.91 [5.48±0.41]	1.18
PM2:Y6	3.51 [3.28±0.25]	4.62 [4.29±0.25]	1.32
PBFTz:Y6	1.38 [1.24±0.13]	4.19 [3.82±0.55]	3.02

a) the hole-only devices with the structure of ITO/PEDOT:PSS/polymer donor/MoO_3/Ag and the electron-only devices with the structure of ITO/ZnO/active layer/PFN-Br/Ag according to the SCLC model. b) the statistical values in square bracket are the average mobilities obtained from 4 $J^{1/2}$-V plots.

Supplementary Note:

Synthesis of PM6:

BDT-TF monomer (0.3 mmol, 282 mg) and bromide monomer of BDD (0.3 mmol, 230 mg) were dissolve in toluene (10 mL). $Pd(PPh_3)_4$ (15 mg) was added into the mixtures after being flushed with argon for ten minutes. Then, the reaction mixtures were purged with argon for another 15 min. The reactions of eight batches of PM6 were stirred at 110°C for about 6 h. The polymers were precipitated in methanol (100 mL) and filtrated. The dried precipitates were purified by flash silica gel column chromatography by using chloroform as eluent. The polymer was then precipitated in methanol (60 mL) and dried under vacuum for 24 h before use.

Synthesis of PM1:

BDT-TF monomer (0.3 mmol, 282 mg) and bromide monomers of BDD (0.24 mmol, 184 mg)and TTz (0.06 mmol, 41 mg) were dissolve in toluene (10 mL). $Pd(PPh_3)_4$ (15 mg) was added into the mixtures after being flushed with argon for ten minutes. Then, the reaction mixtures were purged with argon for another 15 min. The reactions of eight batches of PM1 were stirred at 110°C for about 6 h. The polymers were precipitated in methanol (100 mL) and filtrated. The dried precipitates were purified by flash silica gel column chromatography by using chloroform as eluent. The polymer was then precipitated in methanol (60 mL) and dried under vacuum for 24 h before use. ^{1}H NMR (400 MHz, CD_2Cl_2) δ 7.20-6.08 (m, 7.68H), 2.75 (d, 2H), 2.53-1.80 (m, 6H), 1.14 (s, 4H), 0.78 (d, 34H), 0.59-0.20 (m, 24H). Anal. calcd for $C_{66.8}H_{72.4}F_2N_{0.4}O_{1.6}S_8$: C=66.84%, H=6.04%, N=0.47%, S=21.35%. Found: C= 67.01%, H= 6.31%, N=0.39%, S=21.57%.

Synthesis of PM2:

BDT-TF monomer (0.3 mmol, 282 mg) and bromide monomers of BDD (0.15 mmol, 115 mg)and TTz (0.15 mmol, 103 mg) were dissolve in toluene (10 mL). $Pd(PPh_3)_4$ (15 mg) was added into the mixtures after being flushed with argon for ten minutes. Then, the reaction mixtures were purged with argon for another 15 min. The reaction was stirred at 110 °C for 7 h. The polymers were precipitated in methanol (100 mL) and

filtrated. The dried precipitates were purified by flash silica gel column chromatography by using chloroform as eluent. The polymer was then precipitated in methanol (60 mL) and dried under vacuum for 24 h before use. The yields are around 75%. ^{1}H NMR (400 MHz, CD_2Cl_2) δ 7.18-6.25 (m, 7H), 2.99-1.77 (m, 8H), 1.11 (d, 4H), 0.78 (d, 32H), 0.35 (d, 24H). Anal. calcd for $C_{65}H_{75}F_2N_1O_1S_8$: C=66.15%, H=6.36%, N=1.19%, S=21.71%. Found: C=65.95%, H=6.29%, N=1.05%, S=21.77%.

Synthesis of PBFTz:

BDT-TF monomer (0.3 mmol, 282 mg) and TTz (0.3 mmol, 206 mg) monomerwere dissolve in toluene (10 mL). $Pd(PPh_3)_4$ (15 mg) was added into the mixtures after being flushed with argon for ten minutes. Then, the reaction mixtures were purged with argon for another 15 min. The reaction was stirred at 110 °C for 4 h. The polymers were precipitated in methanol (100 mL) and filtrated. The dried precipitates were purified by flash silica gel column chromatography by using chlorobenzene as eluent. The polymer was then precipitated in methanol (60 mL) and dried under vacuum for 24 h before use. ^{1}H NMR (400 MHz, CD_2Cl_2) δ 7.65 (d, 2H), 7.34 (s, 2H), 7.13 (s, 2H), 2.76 (d, 8H), 2.13-2.07 (m, 1H), 1.94 (d, 1H), 1.67 (d, 6H), 1.18 (d, 28H), 0.93-0.73 (m, 24H). Anal. calcd for $C_{62}H_{74}F_2N_2S_8$: C=65.26%, H=6.49%, N=2.46%, S=22.46%. Found: C= 66.72%, H= 6.99%, N=2.13%, S=20.29%.

Supplementary references

1 Jiang, K. *et al.* Alkyl chain tuning of small molecule acceptors for efficient organic solar cells. *Joule* **3**, 1-14 (2019).
