# Supplementary material for: Random terpolymer based on thiophene-thiazolothiazole unit enabling efficient non-fullerene organic solar cells
Source: Nat Commun. 2020 Sep 14;11:4612. doi: 10.1038/s41467-020-18378-9 (PMC7490407; doi:10.1038/s41467-020-18378-9)
Supplement: Supplementary file 3 — Reporting Summary [file 41467_2020_18378_MOESM3_ESM.pdf]

nature research

Corresponding author(s): DBPR NCOMMS-20-04779A

Solar Cells Reporting Summary

Nature Research wishes to improve the reproducibility of the work that we publish. This form is intended for publication with all accepted papers reporting the characterization of photovoltaic devices and provides structure for consistency and transparency in reporting. Some list items might not apply to an individual manuscript, but all fields must be completed for clarity.

For further information on Nature Research policies, including our data availability policy, see Authors & Referees.

ü Experimental design

Please check: are the following details reported in the manuscript?

1. Dimensions

Item	Yes/No	Response
Area of the tested solar cells	[x] Yes [ ] No	0.04 cm^2
Method used to determine the device area	[x] Yes [ ] No	The device area is calculated by multiplying the width of ITO electrode and metal electrode.

2. Current-voltage characterization

Item	Yes/No	Response
Current density-voltage (J-V) plots in both forward and backward direction	[ ] Yes [x] No	Difference between forward and backward direction was not observed for organic solar cells, thus backward direction scan was not applied in this system.
Voltage scan conditions<br>*For instance: scan direction, speed, dwell times*	[x] Yes [ ] No	From -0.10 V to 1.0 V with 0.01 interval, 10 ms delay.
Test environment<br>*For instance: characterization temperature, in air or in glove box*	[x] Yes [ ] No	The test environment at home lab was in glove box and at room temperature. The device tested at NIM was encapsulated with epoxy. And the test condition at NIM comply to IEC 60904-1 2006, as indicted in the responding test report.
Protocol for preconditioning of the device before its characterization	[ ] Yes [x] No	There is no other protocol for preconditioning before characterization.
Stability of the J-V characteristic<br>*Verified with time evolution of the maximum power point or with the photocurrent at maximum power point; see ref. 7 for details.*	[ ] Yes [x] No	This manuscript is mainly focused on the new materials and device performance, and the stability measurement will be considered in the following studies.

3. Hysteresis or any other unusual behaviour

Item	Yes/No	Response
Description of the unusual behaviour observed during the characterization	[ ] Yes [x] No	No. There is no hysteresis or any other unusual behaviour.
Related experimental data	[ ] Yes [x] No	*Explain why this information is not reported/not relevant.*

4. Efficiency

Item	Yes/No	Response
External quantum efficiency (EQE) or incident photons to current efficiency (IPCE)	[x] Yes [ ] No	EQE is provided.
A comparison between the integrated response under the standard reference spectrum and the response measure under the simulator	[x] Yes [ ] No	The error is less than 5% between the measured Jsc and calculated Jsc from EQE.
For tandem solar cells, the bias illumination and bias voltage used for each subcell	[ ] Yes [x] No	This manuscript is not relevant to tandem solar cells.

5. Calibration

Item	Yes/No	Response
Light source and reference cell or sensor used for the characterization	[x] Yes [ ] No	Yes, all of these information has been included in method.

Confirmation that the reference cell was calibrated and certified

- [x] Yes
- [ ] No

Yes, all of these information has been included in method.

Calculation of spectral mismatch between the reference cell and the devices under test

- [x] Yes
- [ ] No

Yes, all of these information has been included in method.

6. Mask/aperture

Size of the mask/aperture used during testing

- [x] Yes
- [ ] No

2.636 mm^2

Variation of the measured short-circuit current density with the mask/aperture area

- [x] Yes
- [ ] No

When we use mask, the Voc and Jsc are a little lower, the FF is higher, the power conversion efficiency is equivalent compared with no mask.

7. Performance certification

Identity of the independent certification laboratory that confirmed the photovoltaic performance

- [x] Yes
- [ ] No

Yes, we confirmed the photovoltaic performance from independent certification laboratory, and the copy of the certificate was included in the Supplementary Information.

A copy of any certificate(s)
Provide in Supplementary Information

- [x] Yes
- [ ] No

See supplementary information

8. Statistics

Number of solar cells tested

- [x] Yes
- [ ] No

At least 10 cells.

Statistical analysis of the device performance

- [x] Yes
- [ ] No

We show the statistical analysis of the device performance in device performance part in manuscript.

9. Long-term stability analysis

Type of analysis, bias conditions and environmental conditions
For instance: illumination type, temperature, atmosphere humidity, encapsulation method, preconditioning temperature

- [ ] Yes
- [x] No

No. This manuscript is mainly focused on the new materials and device performance, and the stability measurement will be considered in the following studies.
